# Cutting through the noise: A narrative review of Alzheimer's disease plasma biomarkers for routine clinical use

**DOI:** 10.1016/j.tjpad.2024.100056

**Published:** 2025-01-14

**Authors:** M. Schöll, A. Vrillon, T. Ikeuchi, F.C. Quevenco, L. Iaccarino, S.Z. Vasileva-Metodiev, S.C. Burnham, J. Hendrix, S. Epelbaum, H. Zetterberg, S. Palmqvist

**Affiliations:** a Department of Psychiatry and Neurochemistry, Institute of Neuroscience and Physiology, The Sahlgrenska Academy, University of Gothenburg, Gothenburg, Sweden; b Wallenberg Centre for Molecular and Translational Medicine, University of Gothenburg, Gothenburg, Sweden; c Dementia Research Centre, Queen Square Institute of Neurology, University College London, London, UK; d Department of Neuropsychiatry, Sahlgrenska University Hospital, Mölndal, Sweden; e French Institute of Health and Medical Research (Inserm), Paris, France; f Niigata University Brain Research Institute, Niigata, Japan; g Eli Lilly and Company, Indianapolis, IN, United States; h Clinical Neurochemistry Laboratory, Sahlgrenska University Hospital, Mölndal, Sweden; i Department of Neurodegenerative Disease, UCL Institute of Neurology, Queen Square, London, UK; j UK Dementia Research Institute at UCL, London, UK; k Hong Kong Center for Neurodegenerative Diseases, Clear Water Bay, Hong Kong, China; l Wisconsin Alzheimer's Disease Research Center, University of Wisconsin School of Medicine and Public Health, University of Wisconsin-Madison, Madison, WI, USA; m Clinical Memory Research Unit, Clinical Sciences in Malmö, Lund University, Lund, Sweden; n Memory Clinic, Skåne University Hospital, Sweden

**Keywords:** Biomarkers, Alzheimer's disease, Plasma, Neurodegeneration, Clinical routine

## Abstract

As novel, anti-amyloid therapies have become more widely available, access to timely and accurate diagnosis has become integral to ensuring optimal treatment of patients with early-stage Alzheimer's disease (AD). Plasma biomarkers are a promising tool for identifying AD pathology; however, several technical and clinical factors need to be considered prior to their implementation in routine clinical use. Given the rapid pace of advancements in the field and the wide array of available biomarkers and tests, this review aims to summarize these considerations, evaluate available platforms, and discuss the steps needed to bring plasma biomarker testing to the clinic. We focus on plasma phosphorylated(p)-tau, specifically plasma p-tau217, as a robust candidate across both primary and secondary care settings.

Despite the high performance and robustness demonstrated in research, plasma p-tau217, like all plasma biomarkers, can be affected by analytical and pre-analytical variability as well as patient comorbidities, sex, ethnicity, and race. This review also discusses the advantages of the two-point cut-off approach to mitigating these factors, and the challenges raised by the resulting intermediate range measurements, where clinical guidance is still unclear. Further validation of plasma p-tau217 in heterogeneous, real-world cohorts will help to increase confidence in testing and support establishing a standardized approach.

Plasma biomarkers are poised to become a more affordable and less invasive alternative to PET and CSF testing. However, understanding the factors that impact plasma biomarker measurement and interpretation is critical prior to their implementation in routine clinical use

## 1 Introduction

### 1.1 General background

Alzheimer's disease (AD) is a neurodegenerative disorder characterized by progressive cognitive, functional, and behavioral impairment [1]. The two pathological hallmarks are the extracellular accumulation of β-amyloid (Aβ) plaques and the intracellular accumulation of neurofibrillary tangles (NFTs) of phosphorylated tau (p-tau) protein [2]. Current understanding is that the accumulation of these proteins – and their interactions – trigger subsequent pathological changes, including neurodegeneration and inflammation; critically, these changes may begin decades before manifesting into clinical impairment [[2], [3], [4]].

AD is a continuum; there are initial pathophysiological changes followed by subtle neuropsychological abnormalities, with subsequent mild and then progressively-more-extensive cognitive and functional impairments [5]. These are called the preclinical, mild cognitive impairment (MCI), and dementia phases of AD [6]. The timespan between the first identifiable pathophysiological changes, captured by biomarkers, and clinically evident symptomatology is understood to be at least years, if not decades [3,4,7].

### 1.2 Unmet needs in AD diagnosis and treatment

At present, there is no standardized AD diagnostic pathway [8]. In high-income countries, it is estimated that over 50 % of patients with dementia are never formally diagnosed; this percentage is likely even larger in low- and middle-income countries (LMIC) [9,10]. To receive a diagnosis, individuals with cognitive symptoms typically undergo some combination of clinical history taking, cognitive assessments, routine laboratory tests, and structural brain imaging [8]. In a primary care setting, most AD cases are diagnosed clinically without confirmation through disease-specific pathophysiological markers, and often on the basis of ruling out other potential causes, although this may differ by geography [11,12]. Postmortem studies of AD patients have found misdiagnosis rates of up to 30 % [8], with a considerable portion of clinically diagnosed patients lacking substantial Aβ or tau pathology [13]. Crucially, a recent study concluded that the accuracy of both primary (PCPs) and secondary care physicians (i.e., dementia specialists) is substantially lower when trying to diagnose clinical AD without access to biomarker information (61 % and 73 % accuracy, respectively, as opposed to 91 % [both care settings] when biomarker information was made available) [14]. Accordingly, international societies have been calling for the development of a standardized diagnostic pathway and emphasizing the need for including biomarkers to enable accurate and early diagnosis [15].

Given the progressive nature of AD, a timely and accurate diagnosis is likely to produce better outcomes [1]. The introduction of positron emission tomography (PET) and cerebrospinal fluid (CSF) biomarkers promoted confidence in diagnostic accuracy and improved healthcare costs for patients with AD [[16], [17], [18], [19], [20]]; blood-based (i.e., plasma) biomarkers are poised to provide even further benefit, by expanding access to biomarker information beyond specialist or research settings [21]. Broadening biomarker access is especially important given the recent advent of treatments capable of slowing disease progression; those that are currently approved and under regulatory review target AD pathology in early phases, aiming to slow clinical decline via anti-Aβ mechanisms of action [22,23]. These include the monoclonal antibodies lecanemab (investigated in the CLARITY AD trial [24,25]), which has to date been approved in the U.S., Japan, China, South Korea, Hong Kong, Israel, the U.K., and the U.A.E., and has recently received a positive opinion from the European Medicines Agency, and donanemab (investigated in TRAILBLAZER-ALZ2^25^), which has been approved in the U.S., Japan, the U.K., the U.A.E., and Qatar. In addition to enhanced accuracy, biomarkers facilitate earlier diagnosis, which enables intervention while patients’ cognitive and functional abilities are still retained to a greater degree; this can substantially improve the effectiveness of novel therapeutics, slowing progression to prolong the time spent in the initial stages of the disease and improving the subsequent outcomes (e.g., better symptom management and quality of life [QoL]) [26,27].

### 1.3 Importance of biomarkers in AD diagnosis

Since 2007, multiple guidelines have pointed to the crucial role of Aβ and tau biomarkers in the accurate diagnosis of AD, both for research purposes [[28], [29], [30], [31], [32], [33], [34], [35]] and, more recently, clinical practice [15,36,37]. Plasma biomarkers have received their own appropriate use recommendations (AUR) or guidelines (e.g., [[38], [39], [40]]), which outline the minimum acceptable stipulations.

The Alzheimer's Association [AA]) recently published updated guidelines on the diagnostic criteria and staging of AD [41]. This guidance introduces a new classification system with three broad categories of biomarkers: core AD biomarkers (Aβ and tau), non-specific biomarkers important in AD pathogenesis but also involved in other neuropathological diseases, and biomarkers of common, non-AD co-pathologies [41]. Earlier this year, the Global CEO initiative (CEOi) Minimal Acceptable Performance Consensus also highlighted the potential context of use for plasma biomarkers and provided guidance on implementation across different clinical contexts [39], which is discussed later in this review.

### 1.4 Specific need for plasma biomarkers in AD diagnosis

To maximize the beneficial impact on the patient, both memory specialists and general practitioners must be able to achieve timely and accurate biomarker-supported diagnoses [15]. The confirmatory diagnostic tests currently approved by local authorities are Aβ/tau PET and CSF biomarker assessments; however, these existing tools are infrequently utilized due to their (perceived) invasiveness, cost, and low availability stemming from a lack of necessary equipment/expertise and capacity at the basic care level [8,[42], [43], [44], [45]]. Thus, there is a consensus that validated plasma biomarkers will add significant clinical utility compared with PET or CSF due to the greater overall accessibility (e.g., [46]), allowing for early and repeated testing [47]. However, this is also dependent on the performance, accessibility, and scalability of their associated methodology, as discussed in Section 2A.

While research studies have begun screening with plasma biomarkers prior to confirmation with PET or CSF (e.g., [24,25]), thereby reducing participant burden while accurately identifying patient populations, there are additional considerations for a biomarker test to be appropriate for use in a clinical setting. Such considerations include robustness, scalability, and ease of use and interpretation; these were identified along with automation, head-to-head comparisons, and real-world performance as critical research priorities in the 2022 AA AUR for plasma biomarkers in AD [38].

### 1.5 Currently available plasma biomarker assays for AD

Most currently available AD plasma biomarker assays, and those undergoing development, are for research use only (RUOs) [8]. RUOs do not undergo extensive clinical validation, and their regulatory review is limited; as a result, RUOs are only intended for gathering data (from both research and real-world settings) and supporting product development – they are not validated to support clinical diagnosis [8]. Other commercially available biomarker tests are categorized as laboratory-developed tests (LDT) or in-house devices. If they only undergo clinical validation in a local setting, they are only reliable when used in that setting [8]; however, some LDTs have been validated in multiple cohorts, exhibiting robust performance across different settings (e.g., [14] [[48], [49], [50], [51], [52]],). Lastly, tests categorized as in vitro diagnostic devices/tests (IVDs) undergo extensive clinical validation and full regulatory review, and therefore provide robust and reliable test results [8]. RUOs and LDTs may, with further testing and satisfaction of all regulatory requirements, eventually become IVDs; however, it must be noted that regulatory reviews differ by geography [8].

In recent years, an increasing number of biomarker tests from a variety of therapeutic areas has been marketed directly to clinicians and the general public, who may not necessarily understand the inherent limitations and thus risk misclassifying patients due to inconsistent or misinterpreted results [8]. We summarize the commercially available AD plasma biomarker tests in Table 1, below.

**Table 1:** Commercially available AD plasma biomarkers (as of 16 July 2024).

Assay target	Specific test	Company, Location	Measure	Test Category & Regulatory Status	Platform Type
β-Amyloid	Aβ40 and Aβ42 immunoassays [8,53]	EUROIMMUN, Lübeck, Germany	Aβ40 and Aβ42	RUO	ELISA
	ABtest-IA Immunoassay [11,54]	Araclon Biotech, Zaragosa, Spain	Aβ42/40	LDT, CE mark	ELISA
	Aβ 42/40 ratio Immunoassay [55]	Labcorp, Burlington, North Carolina, USA	Aβ42/40	LDT	CLEIA
	HISCL β-Amyloid 1–42 Assay Kit and HISCL β-Amyloid 1–40 Assay Kit [8,56]	Sysmex Corporation, Kobe, Hyogo, Japan	Aβ40 and Aβ42	IVD (manufacturing and marketing approval obtained in Japan)	CLEIA
	Lumipulse G β-Amyloid 1–40 Plasma immunoassay and Lumipulse G β-Amyloid 1–42 Plasma immunoassay [8,57]	Fujirebio, Tokyo, Japan	Aβ40 and Aβ42	RUO	CLEIA
	Aβ40 and Aβ42 Immunoassay [58,59]	Shimadzu Techno-Research Inc, Kyoto, Japan	Aβ40 and Aβ42	LDT in Japan	IP-MS
Tau	Simoa p-tau181 Advantage V2 Kit [8]	Quanterix, Billerica, Massachusetts, USA	p-tau181	RUO (US FDA Breakthrough Device Designation)	SIMOA
	Simoa p-tau181 Immunoassay [8]	ADx Neurosciences NV, Gent, Belgium	p-tau181	Not disclosed	SIMOA
	Simoa p-tau181 immunoassay [8]	University of Gothenburg, Sweden	p-tau181	Not disclosed	SIMOA
	Lumipulse G pTau 181 immunoassay [8,60]	Fujirebio, Tokyo, Japan	p-tau181	RUO	CLEIA
	S-PLEX p-tau181 kit [8,61]	Meso Scale Discovery, Rockville, Maryland, USA	p-tau181	RUO	ECLEIA
	Lumipulse G pTau 217 immunoassay [62]	Fujirebio, Tokyo, Japan	p-tau217	Undergoing evaluation for IVD status; RUO	CLEIA
	p-tau217 Immunoassay [63]	Labcorp, Burlington, North Carolina USA	p-tau217	LDT	CLEIA (on Fujirebio Lumipulse)
	LucentAD p-tau217 Immunoassay [64]	Lucent Diagnostics, Quanterix, Billerica, Massachusetts, USA	p-tau217	LDT	SIMOA
	p-tau217 immunoassay [8]	Eli Lilly Clinical Diagnostics Laboratory, Indianapolis, Indiana, USA	p-tau217	LDT	SIMOA
	Simoa ALZpath p-Tau-217 Advantage PLUS [65]	ALZpath, Inc., Carlsbad, California, USA	p-tau217	RUO	SIMOA
	Elecsys Phospho-Tau (217P) immunoassay [66]	Roche Diagnostics International Ltd, Rotkreuz, Switzerland	p-tau217	Not disclosed; US FDA Breakthrough Device Designation	ECLEIA
	Single-plex p-tau217 assay [67]	Alamar Biosciences, Fremont, California, USA	p-tau217	RUO	NULISA
	Simoa plasma p217+tau assay [8]	Janssen Research & Development, LLC, Raritan, New Jersey, USA	p-tau217	Not disclosed	SIMOA
Panel	Elecsys Amyloid Plasma Panel [8,67,68]	Roche Diagnostics International Ltd, Rotkreuz, Switzerland	p-tau181 & APOE status	US FDA Breakthrough Device Designation; undergoing validation for IVD status	ECLEIA
	PrecivityAD [8,11,69]	C_2_N Diagnostics, St. Louis, Missouri, USA	Aβ42/40, APOE status, & age	LDT, US FDA Breakthrough Device Designation; CE mark	LC-MS/MS
	PrecivityAD2 [48]	C_2_N Diagnostics, St. Louis, Missouri, USA	%p-tau217, Aβ42/40	LDT	LC-MS/MS
	Simoa Neurology 4-Plex E Advantage Kit [8]	Quanterix, Billerica, Massachusetts, USA	Aβ40, Aβ42, GFAP, & NfL	RUO	SIMOA

Abbreviations: Aβ, β-amyloid; AD, Alzheimer's disease; APOE, apolipoprotein E; CE, Conformité Européene; CLEIA, chemiluminescent enzyme immunoassay; ECLEIA, electrochemiluminescent enzyme immunoassay; ELISA, enzyme-linked immunosorbent assay; FDA, Food and Drug Administration; GFAP, glial fibrillary acidic protein; IVD, in vitro diagnostic device; IP-MS, immunoprecipitation-mass spectrometry; LDT, laboratory developed test; LC-MS/MS, liquid chromatography with tandem mass spectrometry; NULISA, NUcleic acid Linked Immuno-Sandwich Assay; p-tau, phosphorylated tau; RUO, research use only; SIMOA, single molecule array.

## 2 Review methodology

### 2.1 Why focus on p-tau assays?

With the wide range of plasma biomarker assays currently available, it can be challenging to select those that would best suit clinical practice; this review aims to elucidate which targets and platforms are most appropriate for routine clinical use. P-tau is generally considered to be the leading plasma biomarker candidate, as it has most reliably demonstrated, in comparison to approved PET and CSF tests, clinically equivalent or superior performance and robustness in diagnostic accuracy and disease specificity across AD phases [49,[70], [71], [72], [73]]. Plasma p-tau217 has been repeatedly shown to perform better than plasma Aβ42/40 at distinguishing individuals with or without Aβ pathology, whether determined by CSF or Aβ PET [[74], [75], [76], [77]]. This has been attributed to the larger fold change (i.e., greater difference in mean concentrations) in plasma p-tau than Aβ42/40 between individuals with and without Aβ pathology [75]. Despite Aβ42/40 being well-validated and established when collected via CSF, plasma-based Aβ42/40 does not have the same robustness for routine clinical use [75,[78], [79]]. This may be due to peripheral metabolism of Aβ [80] or reflect the need for greater sensitivity and specificity (see Table 2 for definitions) in plasma biomarkers, as plasma protein concentrations are over 100-fold lower than corresponding CSF levels [81].

**Table 2:** Definitions of relevant technical terminology.

Term	Definition	Clinical Applications
Clinical Performance	Concordance or agreement of the plasma biomarker with a given reference standard within an intended use population.	Ability to detect AD pathology in early symptomatic (MCI and mild dementia) AD.
Robustness	Quality of (clinical/diagnostic) performance not being affected by small variations introduced through pre-analytical, analytical, batch-to-batch, and lot-to-lot variability.	Reduced risk of misclassifying patient due to variance introduced in testing conditions.
Scalability	Potential for a platform and the associated plasma test to be scaled up to accommodate high volume testing.	Enabling the high-throughput delivery of reliable results with a fast turnaround time.

Abbreviations: AD, Alzheimer's disease; MCI, mild cognitive impairment.

We further limited our most detailed evaluations to those studies that examined p-tau217 or p-tau181, as these biomarkers are furthest in development. While interesting and necessary for a full understanding of the AD disease state, other p-tau biomarkers require further investigation (see Future Directions).

### 2.2 Aβ PET as the preferred reference standard for Aβ confirmation

Historically, the standard of truth for assessing Aβ pathology in potential AD patients has been postmortem evaluation [13]. While this has remained the gold standard, the field has since adopted Aβ PET for diagnostic assessment, as molecular neuroimaging techniques have high diagnostic accuracy. We therefore elected to primarily review studies on plasma biomarkers where Aβ PET was utilized as the reference standard. We included Aβ PET determined through visual read or quantitation, expressed in standardized uptake value ratios (SUVr) and/or centiloid units. However, considering the high concordance of CSF Aβ markers (i.e., CSF Aβ42/40) with Aβ PET [[82], [83], [84], [85]] and further that, in certain countries, the infrastructure makes access to PET challenging, we also included relevant studies where validated CSF Aβ biomarker tests were used as reference standards.

### 2.3 Search strategy & inclusion/exclusion criteria

We obtained the information reviewed herein through keyword and hand searches on PubMed for scientific publications and presentations published between January 2018-October 2024, along with the information available on manufacturer websites. We used the search terms “(Alzheimer) AND (diagnosis) AND (p-tau217 OR p-tau181 OR p-tau231 OR Aβ40/Aβ42 OR Aβ42/Aβ40 OR Aβ40:Aβ42 OR Aβ40:Aβ42)” in combination with either (a) “(sensitivity OR specificity OR AUC OR NPA OR PPA OR NPV OR PPV)”, (b) “(tau PET OR amyloid PET OR CSF) AND (clinical robustness OR total allowable error OR clinical utility)”, (c) “(regulatory status OR assays in development OR RUO OR laboratory developed test OR IVD OR OPA)”, (d) “(limitations OR implementation OR availability OR accessibility OR ease of use OR interpretability)”, or (e) “(patient subtype OR patient hesitancy)”. Following the initial search, we manually screened the results to remove any publications that (a) were meta-analyses, (b) had a sample size of fewer than *n* = 50/group, (c) used a reference standard other than Aβ PET, CSF Aβ42/40, or CSF p-tau181/Aβ42 ratio unless postmortem neuropathology was also utilized, (d) utilized an assay not close enough (within approximately 5 years) to commercial availability, or (e) did not focus on an early symptomatic AD population. Additional relevant studies were manually added as appropriate, for an ultimate total of 92 studies included.

## 3 Technical considerations for plasma biomarker implementation

### 3.1 Background and definitions

An assessment comprises three basic components: the analytes being measured, the instrument or platform used to obtain the measurement, and the criteria used for interpreting the measurement. A novel assessment can only be declared fit-for-purpose in its planned context of use after it has been demonstrated that the measurements obtained are relevant, reliable, interpretable, and statistically sensitive to meaningful change. While the regulatory requirements of what comprises adequate evidence vary by geography, there are multiple technical and clinical aspects that can globally influence these factors. These include clinical performance, clinical robustness, standardization, and scalability, all of which impact the implementation and ease-of-use of a diagnostic test (see Table 2). The technical considerations are discussed below. Refer to Table 3 for definitions of relevant terminology in describing clinical performance [13].

**Table 3:** Definitions of statistical terms used to assess the performance of a diagnostic test [13].

Term	Conceptual Definition	Functional Definition
Sensitivity	Test ability to identify presence of disease marker; results not affected by population.	Proportion of positive SoTs for which the test is positive. TP/(TP+FN).
Specificity	Test ability to identify absence of disease marker; results not affected by population.	Proportion of negative SoTs for which the test is negative. TN/(TN+FP).
Positive Percentage Agreement (PPA)	Test ability to recognize presence of pathology as identified by surrogate SoT; results not affected by population	Same as sensitivity
Negative Percentage Agreement (NPA)	Test ability to recognize absence of pathology as identified by surrogate SoT; results not affected by population.	Same as specificity
Positive Predictive Value (PPV)	Test ability to separate TPs from FPs within a specific population.	Proportion of positive test results that are positive SoTs in a specific population. TP/(TP+FP).
Negative Predictive Value (NPV)	Test ability to separate TNs from FNs within a specific population.	Proportion of negative test results that are negative SoTs in a specific population. TN/(TN+FN).
Area Under the ROC Curve (AUC)	Single scalar value that measures the overall performance of a binary classifier (e.g., a diagnostic test)	Different methods exist; a common approach utilizes the trapezoidal rule, in which the area under the ROC curve is divided into trapezoids – vertical lines at the FP rate values and horizontal lines at the TP rate values – and computed by summing the areas of the trapezoids [86]. An AUC of 0.5 may be considered chance, while 1.0 would indicate perfect classification.
Accuracy or OPA	Measure of biomarker test accuracy (proportion true/all)	(TP+TN)/(TP+FP+TN+FN)

Note that the metrics for each term range between 0 % to 100 % (or 0 to 1), with higher values indicating better performance.

Abbreviations: AUC, area under the curve; FN, false negative; FP, false positive; NPA, negative percent agreement; NPV, negative predictive value; OPA, overall percent agreement; PPA, positive percent agreement; PPV, positive predictive value; ROC, receiver operating characteristic; SoT, standard of truth; TN, true negative; TP, true positive.

### 3.2 Standardization and scalability

Differences in the platforms and materials utilized are important to note when reporting the results of biomarker testing, as suitable methodology may depend on clinical setting [38]. The associated platform type is especially important when considering a biomarker test for routine clinical use, as the platform type may introduce limitations to test scalability. Certain platforms may not have a high throughput and may have a slower turnaround time and/or specific infrastructure or user requirements, which could make adoption in a routine clinical setting more challenging. One crucial factor that affects the standardization and scalability of a test is whether the platform is automated or requires manual handling. Although not comprehensive, evaluations of the fully automated Elecsys® and Lumipulse® platforms indicate that inter- and intra-laboratory coefficients of variance (CVs) are fairly low [87,88]. Manually operated platforms are known to exhibit higher pre-analytical (sample handling) and analytical (inter-laboratory, inter-batch, etc.) variability than fully automated platforms [8,[89], [90]]. Such variability previously hampered the standardization of universal thresholds (cutoffs) for CSF-based diagnosis, as defining and addressing analytical sources of variability is complex [87].

While ultra-sensitive research-grade assays are suitable for single-batch analysis, their introduction into routine clinical use would result in either higher-than-optimal variability in test results or intensive manual labor being required [91]. In addition to reducing analytical variability, fully automated platforms can test samples in larger batches and require less technical expertise, thereby reducing human error, turnaround time, and inter-batch variability, while simultaneously helping to increase testing throughput and potentially also reduce costs.

None of the tests of plasma p-tau217 that utilize commercially available, fully automated platforms for the assessment of AD pathology are yet available as full IVDs; however, fully automated biomarker assays are available as RUOs. There are also mass spectrometry (MS)-based methods of plasma biomarker assays that are commercially available as LDTs in the U.S. and Europe (see Table 1). In a recent head-to-head comparison, immunoprecipitation-mass spectrometry (IP-MS) resulted in better diagnostic performance than immunoassays when measuring plasma Aβ42/40 (immunoassay AUC ranged between 0.69–0–78; AUC=0.86 for the best performing MS-based method) [92]. However, it is likely that immunoassays, which can be performed on fully automated platforms, could be easier and associated with lower costs than MS-based methods to implement in the clinical chemistry laboratories that serve primary care [38]; IP-MS is less scalable [8], due to being associated with more advanced equipment and trained personnel. Therefore, consideration must be given to the needs of the specific clinical context in which the test would be implemented, to identify the appropriate platform.

### 3.3 Clinical performance

One crucial technical consideration is determining the clinical performance of a test and whether it fits the intended use population. The clinical performance of available biomarker assays is assessed via the concordance with a standard of truth; relative to the standard of truth, study authors report metrics such as NPA/NPV and PPA/PPV, AUCs, or other measures estimating assay performance (see Tables 2 and Table 3 for definitions). While AUC is an important measure of agreement with the standard of truth and therefore indicative of the assay's potential for use in diagnostic assessment, the value of this information is limited without the further-advanced analyses of PPA/NPV and NPA/PPV, which consider both the intended use of the assay as well as the prevalence of the patient population. For example, with a low pretest probability of AD (i.e., low prevalence), such as in cognitively unimpaired individuals or patients with clinical syndromes that are atypical for AD, even a high performing test will exhibit a low PPV [38,93]. PPA and NPA can effectively reflect the sensitivity and specificity respectively, when the reference standard is the best available surrogate. However, it should be noted that the terms ‘sensitivity’ and ‘specificity’ are meant to only refer to analyses against a reference/gold standard of truth (i.e., postmortem evaluation of pathology); our review of the literature confirmed that these terms are frequently used out of context. Importantly, accurate prevalence estimates are vital for ensuring accurate PPV and NPV calculations.

Plasma p-tau181 has shown a high concordance with Aβ PET positivity (AUC=0.88) in different cohorts (TRIAD, BioFINDER-2) and can furthermore differentiate AD from vascular dementia (AUC=0.92), progressive supranuclear palsy or corticobasal syndrome (AUC=0.88), behavioral variant frontotemporal dementia/primary progressive aphasia (AUC=0.83), and Parkinson's disease or multiple systems atrophy (AUC=0.82) [94]. This and other studies not only demonstrate that plasma p-tau181 can identify patients with Aβ pathology, but also implies that it is an AD-specific biomarker that can differentiate between other neurodegenerative diseases [[94], [95], [96], [97]].

While plasma p-tau181 has demonstrated high performance in identifying Aβ pathology and the ability to distinguish AD from other neurodegenerative diseases [94,95], it has also been found to exhibit concordance with tau PET. Plasma p-tau181 has been shown to differentiate tau PET+ and tau PET- individuals at different disease (PET-based Braak) stages (PET-based Braak I-II, AUC=0.83; PET-based Braak III-IV, AUC=0.85; PET-based Braak V-VI, AUC=0.85) [94].

Other plasma p-tau isoform biomarkers have been investigated as more promising indicators of AD pathology, specifically p-tau217, but also p-tau231 and p-tau205 [70,[98], [99], [100]]. Understanding the differences in performance and robustness between these biomarkers is key to determining their suitability for specific uses. Round-robin studies, used to test for interlaboratory variation, and head-to-head studies, which directly compare tests to one another, are critical for highlighting these differences. For example, in their head-to-head study comparing plasma p-tau181, p-tau217, and p-tau231, Janelidze et al. [92] found that, regardless of assay type (mass spectrometry or immunoassay), p-tau217 outperformed all other examined p-tau biomarkers in the detection of Aβ pathology (standard of truth: CSF Aβ42/40; AUC=0.86–0.95) [101], a replication of findings from previous work [95]. In addition, all p-tau217 markers assessed were found to outperform other p-tau tests at distinguishing MCI patients who progressed to AD dementia during follow-up (mean follow up=4.9 years) from those who did not (AUC=0.87–0.93) [101]. Furthermore, when another study compared plasma p-tau217 to plasma p-tau231, p-tau181, Aβ42/40, glial fibrillary acidic protein (GFAP), and neurofilament light (NfL), p-tau217 was found to be the only biomarker that changed longitudinally dependent on Aβ pathology and was associated with both preclinical and symptomatic stages [74]. A separate study comparing plasma p-tau181, p-tau231, and p-tau217 also found that plasma p-tau217 outperformed plasma p-tau181 and plasma p-tau231 in identifying individuals with AD pathology [72].

#### 3.3.1 Plasma p-tau217 concordance with Aβ pathology and prediction of cognitive decline

Plasma p-tau217 has demonstrated a high concordance with Aβ pathology whether the standard of truth is Aβ PET [43,70,72,95,104], CSF Aβ42/40,^102^, or a combination of both [103]. Regardless of the assay type, across these studies, p-tau217 reliably differentiated individuals with AD from those with other neurodegenerative diseases as well as healthy controls from individuals with MCI. The accuracy of plasma p-tau217 has also been recently demonstrated as comparable to FDA-approved CSF ratios (Aβ42/40 and p-tau181/Aβ42) when using Aβ PET as the reference standard [49].

Furthermore, p-tau217 can predict future conversion from MCI to dementia, demonstrating an ability to reflect changes across the different phases of AD [102,[104], [105]]. This is further supported by studies reporting a Aβ-dependent sequential increase in plasma p-tau217 levels that is associated with both cognitive impairment and neurodegeneration according to disease severity, from Aβ- cognitively unimpaired individuals all the way through to Aβ+ patients with AD dementia [43,104]. A recent longitudinal study of older participants who were cognitively unimpaired at baseline identified plasma p-tau217 as the best overall predictor of future cognitive decline [106].

#### 3.3.2 Plasma p-tau217 concordance with CSF p-tau217

A recent study found plasma (AUC=0.91) and CSF (AUC=0.94) p-tau217 to be indistinguishable in their performance identifying Aβ pathology, as defined by Aβ PET alone or in combination with tau PET [72]. Conversely, plasma p-tau181 (AUC=0.76) and plasma p-tau231 (AUC=0.82) were found to have a much lower performance than their CSF-based counterparts (p-tau181 AUC=0.87, p-tau231 AUC=0.95) [72]. Other studies reviewed herein concurred no significant difference between the diagnostic performance of plasma and CSF p-tau217 [43,70,76]. Taken together, these findings indicate that plasma p-tau217 can be considered a highly comparable alternative to CSF p-tau217 testing.

#### 3.3.3 Plasma p-tau217 concordance with tau PET

Similar to p-tau181, p-tau217 has also demonstrated high agreement with tau PET, using different radiotracers [70,76,107]. However, when compared with plasma p-tau181, plasma p-tau217 has demonstrated superior performance in identifying tau PET positivity [43,95,107]. Antemortem plasma p-tau217 levels have been shown to significantly correlate with the postmortem density of cortical tau-containing NFTs (Spearman ρ = 0.64, *P* < 0.001), demonstrating a potentially closer relationship of plasma p-tau217 than plasma p-tau181 with tau, particularly NFTs [76]. Another study found a strong correlation between plasma p-tau217 and tau PET (acquired with 18F-flortaucipir-PET) (*r* = 0.61, *P* < 0.001) especially in the temporo-parietal and dorsolateral frontal regions, with regional PET-plasma associations differing according to Aβ burden [108].

Although the consensus from the literature appears to demonstrate a high performance in distinguishing tau PET+ from tau PET- individuals, especially in those with Aβ pathology, there are limitations to using p-tau217 for tau PET-based staging. According to Woo and colleagues (2023), using only plasma p-tau217 resulted in a PPA of 78 % and NPA of 84 % (Cohen's Kappa = 0.62, 95 % confidence interval = 0.43/0.80); however, the addition of plasma NTA-tau (N-terminal tau) along with plasma p-tau217 resulted in the highest agreement with tau PET-based staging, with a PPA of 92 % and NPA of 81 % (Cohen's Kappa = 0.74, 0.57/0.90) [107].

Taken together, the literature indicates a strong relationship between plasma p-tau217 and AD pathology, indicating the ability of plasma p-tau217 to potentially replace confirmatory Aβ CSF and PET testing now that sufficient analytical robustness has been established. However, due to discrepancies in the literature, it appears that the relationship with tau – particularly the clinical performance in tau-based staging – warrants further research. For example, in individuals with autosomal dominant AD, plasma p-tau217 begins increasing 20 years before the onset of MCI and becomes abnormal earlier than tau PET [71]. However, there is also a known time gap between tau protein accumulation as seen postmortem and the in vivo tau PET signal [109], which impacts their association with plasma p-tau217.

### 3.4 Clinical robustness

One of the primary challenges in developing robust AD plasma biomarker assays is the large overlap in plasma biomarker levels between the Aβ+ and Aβ- populations. For example, the well-established and validated biomarker Aβ42/40 is only reduced by approximately 10 % in the plasma of Aβ+ individuals, whereas CSF Aβ42/40 is reduced by 43 % in Aβ+ individuals [79]. One likely explanation for this phenomenon is that the reduction in plasma Aβ42/40 occurs on top of Aβ that is already present, because extracerebrally-produced Aβ is not affected by brain amyloid pathology [38]. This results in a potential risk for misclassification if random error or bias is introduced in the measurement.

Random error may be introduced in multiple ways, which can be minimized but not entirely avoided. These errors may be biological, such as intra-individual variability of biomarker levels in the blood, analytical due to intra- and inter-assay variability between different labs, instruments, or reagent lots, or pre-analytical due to variation in sample collection and handling methodology [110]. A study comparing plasma Aβ42/40, p-tau181, NfL, and GFAP found that inter-assay variations significantly impacted performance for Aβ42/40 but not p-tau181, and that p-tau181 performance was not affected even after introducing maximal variations of up to 20 %, indicative of the robustness of p-tau181 as a plasma biomarker [78].

Pre-analytical sample handling is another large contributing factor to random error. Plasma p-tau response to centrifugation and freeze-thaw cycling has been shown to differ across assays, and the associated protocols also differ between assays [[110], [111], [112], [113]]. Currently existing assays can vary drastically in classifying Aβ status depending on the assay type (mass spectrometry or immunoassay), the antibody, or the reagents used [39]. These are all important considerations when aiming to establish universal pre-analytical guidelines for sample handling.

Both plasma p-tau181 and p-tau231 have been shown to exhibit lower fold changes than their CSF counterparts, which could introduce misclassification from plasma samples. In contrast, both CSF and plasma p-tau217 have similar degrees of overlap between the Aβ+ and Aβ- populations and similar effect sizes, as well as larger fold changes than CSF p-tau181 and p-tau231. The small difference in fold change between plasma p-tau217 and CSF p-tau217 implies that the performance between plasma p-tau217 and CSF p-tau217 may be interchangeable [72].

Test-retest variability has been found to be lowest when using plasma Aβ42/40, and markedly higher with plasma p-tau217; however, due to the considerably larger fold change in the latter, the higher test-retest variability is less of a concern [70]. Cullen and colleagues [79] investigated changes in test AUCs when “noise” or random error was added and found that plasma Aβ42/40 had the lowest test-retest variability (4.1 %), followed by p-tau217 at 20 % and GFAP at 25 % variability [79]. In summary, despite the higher test-retest variability, plasma p-tau217 was the least affected by random error and the highest performing single biomarker in detecting Aβ positivity [70,79].

## 4 Clinical considerations for plasma biomarker implementation

The previous section discussed the clinical utility of plasma biomarkers and the potential for expanding biomarker-based AD diagnosis to non-specialist centers and LMICs. However, to ensure patients receive both a timely and accurate diagnosis, relevant clinical factors such as the care environment, comorbidities, concomitant medications, and potential confounding factors like sex, race, and ethnicity need to be considered prior to the implementation of plasma p-tau assays in routine clinical use. The confounding effects of these factors on p-tau concentrations also need to be related to the effect size of the biomarker change, as induced by the pathology the biomarker is thought to reflect; for instance, Aβ-related tau pathology is thought to increase plasma p-tau217 concentrations by 200–700 % (e.g., [76]). Previous plasma biomarkers have primarily been validated retrospectively, using well-characterized bio-banked samples, which may not reflect the extent of variability encountered in the clinic. To validate a plasma biomarker assay for use in a clinical setting, studies must be completed in naturalistic populations and in settings reflective of real-world practice [8].

Palmqvist and colleagues [14] recently compared physicians’ approximation of ‘likely clinical AD diagnosis’ (determined without the use of biomarkers) at the primary (*n* = 515) and secondary (*n* = 698) care levels versus using a liquid chromatography with tandem mass spectrometry (LC-MS) plasma p-tau217 test [14]. The LC-MS test derived %p-tau217 (the ratio of p-tau217 to non-phosphorylated tau217, multiplied by 100) as well as plasma Aβ42/40 (collectively, the Amyloid Probability Score-2 [APS2]) to detect AD pathology [14]. When using *A*+ (defined using CSF Aβ42/40 [Lumipulse, FDA approved cutoff] or, in a subset [*n* = 82], Aβ PET visual read) and *T*+ (CSF p-tau217) criteria as the reference standard after consensus discussion, the authors found that PCP diagnostic accuracy was only 61 % (53–69 %) [14]. However, when provided APS2 information, diagnostic accuracy increased to >90 % [14]. Similar improvement was seen if using only %p-tau217 [14]. The authors did not compare the performance of %p-tau217 to p-tau217 alone [14]; however, this has been done previously [114]. An increase in diagnostic performance was also observed at the secondary care level, as the diagnostic accuracy of dementia experts was initially 73 % (68–79 %) versus >90 % with plasma biomarker analysis [14]. Despite differences between the two patient populations (primary care patients were older, less educated, and exhibited a higher prevalence of comorbidities), performance was comparable between groups [14]. Interestingly, the authors did not observe a difference in the prevalence of AD pathology between primary and secondary care (approximately 50 % in both settings) [14]. This is an important observation as prevalence or pre-test probability should be a consideration when determining the context of use of a plasma biomarker test [39]. However, it is worth noting that both the primary and secondary care cohorts were recruited within the BioFINDER study (BioFINDER Primary Care; BioFINDER Memory Clinic; BioFINDER2) in southern Sweden, and the prevalence of AD pathology may differ between primary and secondary care settings across different regions.

### 4.1 Comorbidities

Renal function, if not considered, may induce interpretation errors in the assessment of p-tau biomarker levels [115]. Chronic kidney disease (CKD) is associated with higher concentrations of plasma p-tau biomarkers, which is thought to be due to reduced protein clearance from the blood because of poor kidney function; however, CKD is not associated with an increased risk of dementia and could therefore generate false positives [[116], [117], [118]]. In a recent study by Lehmann and colleagues [119], plasma levels of p-tau181 and p-tau217 were both shown to strongly correlate with creatinine levels and estimated glomerular filtration (eGFR) rates) [119]. However, while the authors observed no impact on p-tau217 performance, the performance of p-tau181 was significantly impacted by renal function [119]. Similarly, when examining the effect of renal function on the performance of plasma p-tau181, Zhang et al. [120] found that the cut-off values for p-tau181 were changed by up to 19 % and 23 % in patients with mild (60–90 ml/min) and moderate (<60 ml/min) eGFR decline, respectively, compared to participants with normal eGFR [120]. Furthermore, a recent study found that the mean difference in plasma p-tau181 levels between patients with and without CKD was greater than the difference between Aβ+ and Aβ- populations (as determined by Aβ PET) [121]. Notably, while p-tau217 levels were also elevated in patients with CKD, the mean difference between those with and without CKD remained smaller than that between the Aβ+ and Aβ- populations [121]. Additionally, another study found that plasma p-tau217 distinguished between Aβ+ and Aβ- patients with CKD with a 4–7 % misclassification rate [116]. Collectively, then, these findings indicate that reference values specific to renal function should be implemented for plasma p-tau181 and suggest that plasma p-tau181 may not be sufficiently robust to serve as a standalone AD pathology marker. By contrast, plasma p-tau217 appears to potentially have the necessary robustness, including in the assessment of patients with MCI and mild dementia, though the effects of renal failure still ought to be considered. Using a ratio or percentage such as plasma %p-tau217 may further mitigate the effects of renal impairment on plasma concentrations [114]; Palmqvist et al. [14] utilized plasma %p-tau217 for this reason, and the test performed well despite a high rate of comorbid CKD (26 % in the primary care cohort) [14]. However, ratios may be subject to skew by other neurological diseases, reducing the specificity for AD pathology [122].

Additionally, in age- and sex- adjusted analyses, Mielke et al. [121] initially linked increased levels of plasma p-tau181 and plasma p-tau217 to stroke, hypertension, and myocardial infarction; however, after excluding CKD patients from analysis, only myocardial infarction remained significantly associated, further underlining the impact of comorbidities in biomarker studies [121]. Notably, while these adjusted analyses did not find a robust association between decreases in plasma p-tau and high BMI until patients with CKD were removed [121], a recent study in the BALTAZAR cohort has reported that high BMI was associated with decreases in both plasma p-tau217 and plasma p-tau181 [117,119].

### 4.2 Sex, race, and ethnicity

Historically, biomarkers have been validated in clinical cohorts with minimal diversity in terms of sex and race/ethnicity, which is not fully representative of real-world clinical settings across different geographies. Further research is needed to understand the potential impact of sex and race/ethnicity in biomarker testing.

Research suggests that, compared to men, women with AD may have higher levels of tau pathology (as measured with either CSF- or PET-based biomarkers), including a greater burden of tau tangles at autopsy, and may experience faster cognitive decline [123]. However, findings have been inconsistent within and across tau isoforms; for example, Mattsson et al. (2016) reported no sex differences in plasma levels of total tau (T-tau, the sum of all tau isoforms, irrespective of phosphorylation status) [124], while Baldacci et al. (2020) reported that females had significantly higher levels of T-tau [125]. A recent study found that, among Aβ+ MCI patients, women exhibited higher rates of incident AD dementia than men, which was associated with increased baseline plasma p-tau181 levels [126]; by contrast, most studies that evaluate plasma p-tau217 report no sex differences in plasma concentrations [127].

Research has also demonstrated that plasma biomarker levels may differ across racial and ethnic groups [39]. However, results remain inconsistent. For example, some research groups have found plasma levels of Aβ42, Aβ40, p-tau181, and NfL to be lower in African American (AA) individuals than white individuals [128], while others did not identify any differences between plasma biomarker levels in AA and non-Hispanic white (NHW) individuals [129]. One study matching AA participants to non-Hispanic white (NHW) participants by age, sex, *APOE* ε4 carrier status, and cognitive status, found that while plasma Aβ42/40 could accurately measure brain amyloidosis across both groups, plasma p-tau181, p-tau231, and NfL performed inconsistently, risking the misdiagnosis of AA individuals [130]. This contrasts with work by Brickman and colleagues (2021), who found that p-tau181 had a higher predictive performance in non-white individuals than white for AD neuropathological but not clinical diagnosis [131]. The inconsistency among reported findings may stem from the difficulty of disentangling race and ethnicity from socioeconomic status, which is itself linked to increased dementia risk [132]. There remains an unmet need for larger, more diverse study populations that validate blood biomarkers in all racial and ethnic groups.

## 5 Guidelines for best practices in plasma biomarker implementation

Work toward implementing plasma biomarkers in clinical practice has begun [39,40]. Ensuring best practice implementation of plasma biomarker testing in the clinic requires the consideration of technical limitations along with the intended use (i.e., triaging or confirmatory; at secondary/tertiary or primary care level). As mentioned alongside technical considerations, the clinical performance (PPA/NPA) of a plasma biomarker test indicates its potential to be used as either a triaging (rule-out AD) or confirmatory (rule-in AD) test. Notably, the Global CEO initiative on Alzheimer's disease has published a consensus on the minimum acceptable performance of plasma biomarkers for clinical use, which is purpose-dependent: if the biomarker is to be used as a triaging test (i.e., before a further confirmatory test), they recommend a minimum sensitivity of >90 % and minimum specificity of >85 % in a primary care setting, with slightly more lenient requirements in secondary care (e.g., >75–85 %) due to better accessibility of confirmatory testing [39]. If the biomarker is to be used as a confirmatory test, the initiative recommends that it perform equivalently to FDA-approved IVD CSF tests, with a minimum of >90 % sensitivity and specificity [39]. However, even with high levels of sensitivity and specificity, the pre-test probability of AD must be taken into consideration at the time of test result interpretation; if the pre-test probability is low, even a highly sensitive and specific test will have a low PPV [39].

### 5.1 A two-cut-off approach

Given that approximately 5–20 % of individuals have a borderline level of Aβ pathology and may thus receive discordant results in repeated testing if only a single cut-off value is utilized, the Global CEOi recommends [39] a two-cut-off approach, which defines three categories of test results: positive, intermediate, and negative [133].

Many labs have developed their own (in-house) two-point cut-off values; however, such lab-specific cut-offs impact the generalizability of the subsequent findings. Additionally, there is currently no standardized approach to presenting the intermediate category. To promote transparency, we recommend that the full 3 (test results – positive, negative, or intermediate) x 2 (true positive or true negative) confusion matrix be reported, to enable centers to conduct their own calculations and avoid ambiguity. Furthermore, both PPV and NPV values should be reported, as well as the intermediate results, to help fully illustrate the test's clinical utility. In the future, these data will help fill the currently unmet need for validated and robust tests with standardized cut-offs that are applicable in a heterogeneous population reflective of the real-world clinic.

Stratifying patients through a low and high cut-off optimized for NPA and PPA, respectively, has been reported to improve test performance [14,70,133] and increase plasma test accuracy in both primary and secondary care settings [14]. Ashton and colleagues [70] established a three-range reference for AD positivity using plasma p-tau217 in the Wisconsin Registry for Alzheimer's Prevention (WRAP) cohort, which they cross-validated in the Translational Biomarkers in Aging and Dementia (TRIAD) and Sant Pau Initiative on Neurodegeneration (SPIN) cohorts [70]. The three-range reference resulted in an improved PPA while maintaining similar NPA; the overall percent agreement was also improved [70]. Similarly, a recent study investigating eligibility for novel anti-amyloid therapies in a cohort from the Butler Memory & Aging Program (MAP) found that a two-cut-off approach was not only feasible in a memory clinic setting, but also slightly increased the diagnostic performance compared to a single cut-off approach [134]. Importantly, a two-step workflow designed in BioFINDER-1 and then validated in BioFINDER-2, also within a memory clinic setting, was found to accurately classify Aβ-PET status and substantially decrease the number of necessary CSF tests [133], suggesting that plasma p-tau217 testing can be a cost-effective approach to detecting AD when the two-cut-off approach is employed. The BioFINDER-1 model was also validated in the TRIAD cohort, indicating that the probability thresholds derived in the study performed well across assays and geographies without the need for re-optimization [133].

### 5.2 The challenge associated with intermediate results

While the two-cut-off approach improves accuracy and may potentially increase cost-effectiveness, it is worth noting that tests with higher variability will produce a higher proportion of intermediate (also known as indeterminate) results, increasing the need for confirmatory PET/CSF testing. Several studies have reported a range in intermediate results; for example, Ashton et al. [70] found an intermediate percentage of between 12.3 % and 22.9 % across cohorts in their study [70], while Howe et al. (2024) found that 24 % of study participants were in the intermediate category [134]. When Brum et al. (2023) applied differing levels of stringency to investigate the effect on overall accuracy and associated reduction in CSF testing, they found that at the highest level of stringency, an accuracy of 92.7 % (95 % CI = 88.9–94.6 %) resulted in 38.8 % of participants requiring confirmatory testing [133]. Meanwhile, Mattsson-Carlgren and colleagues (2023) found that, at a false-negative rate less than 10 %, 43.1 % of participants required further tests to identify high tau PET in Aβ-positive participants [103].

The Global CEOi on AD working group have recommended that a plasma biomarker test result in no more than 15–20 % of patients of a standard clinical population falling into the intermediate category [39]. They have also proposed that intermediate results be approached on a case-by-case basis, with Aβ PET or CSF testing being completed if available; when confirmatory tests are not available, a repeated blood test at a later date may be an appropriate alternative [39]. It is also worth noting that intermediate test results may be reflective of truly intermediate amounts of AD pathology, in which case additional CSF or Aβ PET testing would only confirm the patient is in a transitional pathophysiological phase. A further complication is that, if the plasma assay performance is equivalent to that of CSF/PET biomarkers, further testing may not provide the necessary verification.

## 6 Future directions and conclusions

To summarize, the literature featured in this review demonstrates that plasma biomarkers of AD, particularly plasma p-tau217, exhibit high clinical performance and robustness, and may provide an effective and non-invasive method to test for AD pathology in a routine clinical setting. Adoption of plasma biomarker testing in early-stage AD may especially improve timely access to treatment, which is of greatest benefit at this stage [26,27].

### 6.1 Future directions

Studies have begun reporting equally high diagnostic performance of plasma p-tau217 across primary and secondary care [14]. This suggests that plasma tests could be used to determine which patients should be referred without delay for further evaluation and potential treatment with novel therapies.

In a recent study investigating the clinical utility of plasma p-tau217 for selecting appropriate patients to receive anti-amyloid therapeutics, Howe and colleagues reported high diagnostic accuracy, with a slight increase when using the two-cutoff approach; notably, regardless of the approach used, there remained false positives (i.e., Aβ- individuals who were identified for aducanumab treatment) [134]. However, a false positive in which the patient receives a positive plasma p-tau217 test result but a negative Aβ PET scan may be indicative of an elevated amyloid burden that is (yet) below the threshold for PET detection [135]. The Global CEOi consensus provides helpful guidance in this regard [39], but prospective validation in clinical cohorts from both primary and secondary care settings are still needed. In relation to identifying patients for novel treatment options, another possible future use case for plasma biomarkers could be in monitoring disease progression and/or treatment response, and potentially predicting future disease progression [8]. Such monitoring could be invaluable to clinicians when making treatment decisions. Furthermore, it is not the case that clinicians are merely interested in knowing whether their patient has AD; depending on the symptoms, a negative result on a test with high diagnostic accuracy could be suggestive of a non-AD dementia, which ought to be referred to secondary care.

In addition to those covered in this review, other plasma biomarker assays could have clinical applications along different stages of the AD disease pathway and care continuum. Investigating a panel of other plasma p-tau isoforms such as p-tau205 and p-tau231 could help the field further understand intraindividual differences in tau pathophysiology [136]. The microtubule-binding region (MTBR) of tau containing the residue 243 (MTBR-tau243) is a biomarker of insoluble tau aggregates that has recently demonstrated promising results, although it is still very early in development [137]. As a biomarker closely associated with tau tangle pathology, MTBR-tau243 could help clinicians accurately determine whether their patient's symptoms stem from AD pathology or a co-pathology [137]. While current studies focus on CSF-based analysis of MTBR-tau243, efforts to develop a plasma assay are underway, with preliminary results similar to those observed in CSF [138].

While this review has primarily focused on the implementation of plasma biomarkers for the diagnosis of early-stage AD, another potential future use case for plasma tests could be the detection of AD pathology at the preclinical stage, in order to enable secondary interventions that could slow down and perhaps even one day prevent the disease [[139], [140], [141]]. There are several ongoing clinical trials, such as AHEAD 3–45 (NCT04468659), TRAILBLAZER-ALZ 3 (NCT05026866), and TRAILRUNNER-ALZ 3 (NCT06653153), that are investigating preclinical AD and incorporating plasma tests for Aβ pathology as screening tools or secondary/exploratory outcomes, which highlights the growing importance and potential of this biomarker in identifying preclinical AD. If these trials demonstrate that removing AD pathological burden prior to the emergence of overt symptoms reduces the risk of declining to a status of MCI/dementia, then the plasma biomarkers utilized in these trials could be considered for use in clinical practice with asymptomatic elderly individuals. This secondary prevention scheme could resemble that of many other chronic conditions (e.g., diabetes, hypercholesterolemia, etc.), where identifying an abnormal value on a simple blood test warrants a specific course of treatment, with demonstrated individual and societal benefits. However, the low prevalence of AD pathology when screening cognitively healthy individuals will present a challenge, as even a highly sensitive and specific test will have a reduced PPV under low prevalence conditions [39].

### 6.2 Conclusion

Plasma biomarkers have the potential to serve as a less-invasive alternative for the detection of pathology in early-stage AD. However, multiple technical and clinical factors must be taken into consideration to ensure that both high performance and robustness are retained in routine clinical settings. Fully automated testing platforms produce the least variability and will be necessary for maintaining high performance without sacrificing time and resources. Clinicians must consider the potential impact of patient characteristics on plasma biomarker levels; in particular, that of reduced renal function, which can generate false positives. This is especially relevant to a primary care setting, where patients are likely to be more heterogenous. Standardized two-point cutoff values could help achieve sufficiently high levels of performance as to drastically minimize CSF/PET testing; however, further guidance on interpreting intermediate results is needed. As the field develops and more robust, high-performing blood tests become available with prospectively, clinically validated cutoffs, two-point cutoffs could eventually be replaced with reliable binary cutoffs. In the more distant future, a binary cutoff could replace CSF/PET testing entirely, albeit with lower PPV and NPV. Future validation in real-world cohorts could help establish robust cutoffs for diverse clinical settings, thereby reducing intermediate test results and enabling timely diagnosis and access to novel treatment options.

## CRediT authorship contribution statement

**M. Schöll:** Writing – review & editing, Conceptualization. **A. Vrillon:** Writing – review & editing. **T. Ikeuchi:** Writing – review & editing. **F.C. Quevenco:** Writing – review & editing, Writing – original draft, Supervision, Project administration, Formal analysis, Data curation, Conceptualization. **L. Iaccarino:** Writing – review & editing, Writing – original draft, Conceptualization. **S.Z. Vasileva-Metodiev:** Writing – review & editing, Writing – original draft, Conceptualization. **S.C. Burnham:** Writing – review & editing, Writing – original draft, Conceptualization. **J. Hendrix:** Writing – review & editing, Writing – original draft, Conceptualization. **S. Epelbaum:** Writing – review & editing, Writing – original draft, Supervision, Formal analysis, Conceptualization. **H. Zetterberg:** Writing – review & editing. **S. Palmqvist:** Writing – review & editing, Conceptualization.

## Declaration of competing interest

The authors declare the following financial interests/personal relationships which may be considered as potential competing interests: Frances-Catherine Quevenco reports administrative support, article publishing charges, and writing assistance were provided by Eli Lilly and Company. Michael Schöll reports a relationship with BioArctic AB that includes: funding grants, speaking and lecture fees, and travel reimbursement. Michael Schöll reports a relationship with Roche Diagnostics Corporation that includes: funding grants and speaking and lecture fees. Michael Schöll reports a relationship with Novo Nordisk Inc that includes: speaking and lecture fees. Michael Schöll reports a relationship with Triolabs that includes: speaking and lecture fees. Michael Schöll reports a relationship with Centile Bioscience that includes: equity or stocks. Sebastian Palmqvist reports a relationship with Avid Radiopharmaceuticals Inc that includes: funding grants. Sebastian Palmqvist reports a relationship with Alzheimer's Drug Discovery Foundation that includes: funding grants. Sebastian Palmqvist reports a relationship with Eisai Inc that includes: consulting or advisory and travel reimbursement. Sebastian Palmqvist reports a relationship with Roche Diagnostics Corporation that includes: consulting or advisory and speaking and lecture fees. Sebastian Palmqvist reports a relationship with Eli Lilly and Company that includes: consulting or advisory and speaking and lecture fees. Sebastian Palmqvist reports a relationship with BioArctic AB that includes: consulting or advisory and travel reimbursement. Sebastian Palmqvist reports a relationship with Novo Nordisk Inc that includes: consulting or advisory and travel reimbursement. Agathe Vrillon reports a relationship with A2MCL that includes: funding grants and travel reimbursement. Agathe Vrillon reports a relationship with Bioprojet that includes: funding grants. Agathe Vrillon reports a relationship with Foundation Overcoming Alzheimer that includes: funding grants. Agathe Vrillon reports a relationship with France Alzheimer that includes: travel reimbursement. Takeshi Ikeuchi reports a relationship with Japan Agency for Medical Research and Development that includes: funding grants. Takeshi Ikeuchi reports a relationship with Eisai Inc that includes: speaking and lecture fees. Takeshi Ikeuchi reports a relationship with FUJIREBIO Inc that includes: speaking and lecture fees. Takeshi Ikeuchi reports a relationship with Eli Lilly and Company that includes: speaking and lecture fees. Frances-Catherine Quevenco reports a relationship with Eli Lilly and Company that includes: employment and equity or stocks. Leonardo Iaccarino reports a relationship with Eli Lilly and Company that includes: employment and equity or stocks. Simona Z. Vasileva-Metodiev reports a relationship with Eli Lilly and Company that includes: employment and equity or stocks. Samantha C. Burnham reports a relationship with Eli Lilly and Company that includes: employment and equity or stocks. James Hendrix reports a relationship with Eli Lilly and Company that includes: employment and equity or stocks. Stephane Epelbaum reports a relationship with Eli Lilly and Company that includes: employment and equity or stocks. Henrik Zetterberg reports a relationship with AD Strategic Fund (Alzheimer's Association) that includes: funding grants. Henrik Zetterberg reports a relationship with Alzheimer's Drug Discovery Foundation that includes: funding grants. Henrik Zetterberg reports a relationship with Bluefield Project that includes: funding grants. Henrik Zetterberg reports a relationship with Cure Alzheimer's Fund that includes: funding grants. Henrik Zetterberg reports a relationship with Erling Persson Family Foundation that includes: funding grants. Henrik Zetterberg reports a relationship with EU Joint Programme Neurodegenerative Disease Research that includes: funding grants. Henrik Zetterberg reports a relationship with Hjärnfonden that includes: funding grants. Henrik Zetterberg reports a relationship with Horizon Europe that includes: funding grants. Henrik Zetterberg reports a relationship with National Institute for Health and Care Research UCLH Biomedical Research centre that includes: funding grants. Henrik Zetterberg reports a relationship with Olav Thon Foundation that includes: funding grants. Henrik Zetterberg reports a relationship with Stiftelsen för Gamla Tjänarinnor that includes: funding grants. Henrik Zetterberg reports a relationship with Swedish Research Council that includes: funding grants. Henrik Zetterberg reports a relationship with Swedish State Support for Clinical Research that includes: funding grants. Henrik Zetterberg reports a relationship with UK Dementia Research Institute that includes: funding grants. Henrik Zetterberg reports a relationship with AbbVie Inc that includes: consulting or advisory. Henrik Zetterberg reports a relationship with Acumen Pharmaceuticals, Inc. that includes: consulting or advisory. Henrik Zetterberg reports a relationship with Alector Inc that includes: consulting or advisory. Henrik Zetterberg reports a relationship with Alzinova AB that includes: consulting or advisory. Henrik Zetterberg reports a relationship with ALZPath that includes: consulting or advisory. Henrik Zetterberg reports a relationship with Amylyx Pharmaceuticals Inc that includes: consulting or advisory. Henrik Zetterberg reports a relationship with Annexon Biosciences that includes: consulting or advisory. Henrik Zetterberg reports a relationship with Apellis Pharmaceuticals, Inc that includes: consulting or advisory. Henrik Zetterberg reports a relationship with Artery Therapeutics Inc that includes: consulting or advisory. Henrik Zetterberg reports a relationship with AZTherapies that includes: consulting or advisory. Henrik Zetterberg reports a relationship with Cognito Therapeutics that includes: consulting or advisory. Henrik Zetterberg reports a relationship with CogRx that includes: consulting or advisory. Henrik Zetterberg reports a relationship with Denali Therapeutics Inc that includes: consulting or advisory. Henrik Zetterberg reports a relationship with Eisai Inc that includes: consulting or advisory. Henrik Zetterberg reports a relationship with LABCORP that includes: consulting or advisory. Henrik Zetterberg reports a relationship with Merry Life Biomedical that includes: consulting or advisory. Henrik Zetterberg reports a relationship with NervGen Pharma Corp that includes: consulting or advisory. Henrik Zetterberg reports a relationship with Novo Nordisk Inc that includes: consulting or advisory and speaking and lecture fees. Henrik Zetterberg reports a relationship with Optoceutics that includes: consulting or advisory. Henrik Zetterberg reports a relationship with Passage Bio Inc that includes: consulting or advisory. Henrik Zetterberg reports a relationship with Pinteon Therapeutics Inc that includes: consulting or advisory. Henrik Zetterberg reports a relationship with Prothena Biosciences Inc that includes: consulting or advisory. Henrik Zetterberg reports a relationship with Red Abbey Labs that includes: consulting or advisory. Henrik Zetterberg reports a relationship with reMYND that includes: consulting or advisory. Henrik Zetterberg reports a relationship with Roche Diagnostics Corporation that includes: consulting or advisory and speaking and lecture fees. Henrik Zetterberg reports a relationship with Samumed that includes: consulting or advisory. Henrik Zetterberg reports a relationship with Siemens Healthineers AG that includes: consulting or advisory. Henrik Zetterberg reports a relationship with Triplet Therapeutics Inc that includes: consulting or advisory. Henrik Zetterberg reports a relationship with Wave that includes: consulting or advisory. Henrik Zetterberg reports a relationship with AlzeCure Pharma that includes: speaking and lecture fees. Henrik Zetterberg reports a relationship with Biogen Inc that includes: speaking and lecture fees. Henrik Zetterberg reports a relationship with Cellectricon AB that includes: speaking and lecture fees. Henrik Zetterberg reports a relationship with Eli Lilly and Company that includes: speaking and lecture fees. Henrik Zetterberg reports a relationship with FUJIREBIO Inc that includes: speaking and lecture fees. Henrik Zetterberg reports a relationship with WebMD LLC that includes: speaking and lecture fees. Henrik Zetterberg reports a relationship with Brain Biomarker Solutions in Gothenburg AB that includes: equity or stocks. If there are other authors, they declare that they have no known competing financial interests or personal relationships that could have appeared to influence the work reported in this paper.
